# The Latest Humbug

**Published:** 1861-05

**Authors:** 


THE LATEST HUMBUG.—In the kingdom of Hanover, at the foot of Mount Iberg, is a small town, Grund am Hartz, situated near large pine forests, which is becoming celebrated throughout Germany on account of the *pine-leaf cure*, instituted there in 1855. It is alleged that cases of gout, rheumatism, scrofula, neuralgia, asthma, hysteria, phthisis, and inflammation of the mucous membranes have either been cured or greatly relieved by this mode of treatment. The extent to which it is carried may be seen from the following extract from the *Medical Times and Gazette:* "In cases of muscular rheumatism, pine-leaf vapor-baths are given, and pine-wood cataplasms applied to the suffering parts, which are also rubbed with the essential oil of the pine; besides which, two to four ounces of pine-juice are prescribed once or twice a day; for which, when the bowels are constipated, pine saline water is substituted." In fact, the leaves of the pine, either as a bath, inhalation, or solution, combined with other remedial agents, are used in every case which may present itself at the "kuranstalt." "*Vive la bagatelle!*"

